# Comparison of Short-term Outcomes Between Schneiderian Membrane Perforation and Non-perforation Patients after Simultaneous External Elevation and Implantation

**DOI:** 10.3290/j.ohpd.b5638110

**Published:** 2024-07-30

**Authors:** Jichao Lin, Qianrong Zhou, Yanjun Lin, Wei Bi, Youcheng Yu, Qinglian Wang

**Affiliations:** a Department of Stomatology, Zhongshan Hospital (Xiamen), Fudan University, Xiamen, Fujian Province, China. Conceived and designed the research, wrote the manuscript, read and approved the final manuscript.; b Department of Stomatology, Zhongshan Hospital, Fudan University, Shanghai, China. Carried out the experiments, read and approved the final manuscript.; c Department of Dental Implantology, School and Hospital of Stomatology, Fujian Medical University, Fuzhou, Fujian Province, China. Performed data analysis, wrote the manuscript, read and approved the final manuscript.; d Department of Stomatology, Zhongshan Hospital, Fudan University, Shanghai, China. Revised the manuscript, read and approved the final manuscript.; e Department of Stomatology, Zhongshan Hospital, Fudan University, Shanghai, China. Revised the manuscript, read and approved the final manuscript.; f Department of Stomatology, Zhongshan Hospital (Xiamen), Fudan University, Xiamen, Fujian Province, China. Revised the manuscript, read and approved the final manuscript.; * Jichao Lin and Qianrong Zhou are the first authors and contributed equally to this work.

**Keywords:** dental implantation, implantation complication, maxillary sinus, retrospective study, Schneiderian membrane perforation

## Abstract

**Purpose:**

To compare short-term outcomes between membrane perforation and non-perforation patients after simultaneous external elevation with implantation.

**Materials and Methods:**

In this retrospective observational study, 60 maxillary posterior tooth-loss patients with an insufficient amount of residual bone for direct implantation were enrolled. All patients underwent simultaneous external elevation and implantation, and were divided into perforation and non-perforation groups according to the postoperative Schneiderian membrane status.

**Results:**

Of the 60 patients, 30 cases (35 implants) were assigned to the membrane perforation group, and 30 (44 implants) were allocated to the non-perforation group. There were no statistically significant differences in baseline data (p>0.05). In the perforation group, the mean vertical bone gain (VBG) at 6 and 12 months was 6.02±2.14 mm and 5.37±2.22 mm, resp., compared to 6.78±2.59 mm and 6.42±2.64 mm in the non-perforation group, resp. (both p>0.05). Preoperative median Schneiderian membrane thickness (SMT) in the perforation group was 0.77 mm, which was statistically significantly thinner than the 1.24 mm measure in the non-perforation group (p<0.05); however, there was no statistically significant difference between two groups at 12 months postoperatively (0.80 mm vs 1.25 mm, p>0.05). The marginal bone loss at 1 year after implant restoration in the perforation and non-perforation groups was 0.16±0.10 mm and 0.22±0.12 mm, resp. During postoperative follow-up, the implant survival rate was 100% in the two groups. The incidence of postoperative nasal bleeding in the perforation group was statistically significantly higher compared with that in the non-perforation group (50% vs 16.7%, p<0.05), whereas no statistically significant differences were observed in the incidence of facial swelling, intraoral bleeding, wound dehiscence and acute/chronic sinusitis between the two groups (p>0.05).

**Conclusions:**

Schneiderian membrane perforation after simultaneous external elevation and implantation do not adversely affect short-term clinical and radiographic outcomes.

Posterior tooth loss can impair the ability to chew food, making it less easily digestible and lead to a decreased quality of life. Dental implants have become the “gold standard” for restoring the aesthetics and function of missing teeth in modern dentistry.^24^

Post-extraction in the maxillary posterior region often leads to sinus pneumatisation^15,22^ and vertical alveolar ridge resorption. Internal and external sinus elevation^3,10^ is used, depending on the patient’s condition, with the aim of providing sufficient bone support for implants. External elevation was first described by Boyne and James.^3^ In cases of severe ridge atrophy in the posterior maxilla, patients typically undergo direct sinus augmentation followed by implant placement after 6-9 months and restoration after another 3-4 months.

To shorten the treatment time, simultaneous external elevation and implantation are recommended for patients such that dental implants will have primary stability,^6,7^ and the root tip of the implant can act as a “tent pole” to support the maxillary sinus membrane and maintain the osteogenic space.^5^

Schneiderian membrane perforation is the most common complication during external elevation surgery.^6,11,16,19^ Perforation of the membrane makes a direct communication to the maxillary sinus. Via this communication, the graft material can be scattered into the sinus; however, it can also cause infection or sinusitis.^11^

Smokers are more prone to maxillary sinus perforation; anatomical factors such as Schneiderian membrane thickness,^14^ sinus septa,^12^ and the residual bone^2^ may also favour maxillary sinus perforation. In addition, a history of previous maxillary sinus operation^25^ and the experience of the surgeon can influence the risk of perforation.

In cases where a large perforation is difficult to manage during surgery, the surgeon may choose to forgo further surgical dissection and bone grafting, and wait approximately six months for the maxillary sinus mucosa to heal before attempting a secondary approach of lifting and grafting. However, the literature reports that bone augmentation surgery is delayed in only a very small portion (< 1%) of patients, due to excessively large perforations.^4^ Typically, for perforations ≤ 10 mm in diameter, the mucosa can be dissected away from the sinus floor, at some distance from the perforation to reduce tension and minimise the area of the perforation. After elevating the mucosa around the perforation to the level of the sinus roof, it can be covered with an absorbable collagen membrane.^27^

Recent studies have shown that simultaneous implantation and bone grafting have a high success rate and satisfactory clinical results in solving maxillary sinus perforation.^2,8,32^ However, there are some discrepancies in the results between different studies, and further studies are needed to validate them. In this retrospective study, the short-term outcomes between membrane-perforation and non-perforation patients after simultaneous external elevation and implantation were comparatively analysed, aiming to confirm the applicability of this combined approach in patients with Schneiderian membrane perforation.

## MATERIALS AND METHODS

### Study Design

In this retrospective observational study, patients with maxillary posterior tooth loss and an insufficient amount of residual bone for direct implant repair admitted to our hospital between March 2018 and September 2021 were recruited. All patients were informed about the surgical and restoration procedures. The study was approved by the Medical Ethics Committee of our hospital (B2021-767) and performed in accordance with the Helsinki Declaration (revised in 2008).

### Inclusion and Exclusion Criteria

The included participants were patients 1) with maxillary posterior tooth loss and preoperative residual bone height <6 mm; 2) undergoing dental implant repair; 3) with good oral hygiene; 4) with no perforation or bleeding before surgery; and 5) with normal coagulation function. Patients were excluded if they: 1) had an acute infection; 2) had a maxillary sinus cyst or septum; 3) smoked >10 cigarettes/day; 4) were taking oral bisphosphonates; 5) suffered from systemic diseases, such as heart disease, hypertension, or diabetes mellitus; or 6) had hepatitis B, syphilis, AIDS, or other infectious diseases.

### Surgical Procedures

All surgical procedures were performed by the same surgeon with 20 years of dental implantation experience. The operation was performed under local anesthesia. A horizontal incision was made on the top of the alveolar ridge in the edentulous area, and a vertical incision was created mesiodistally to the implant to form a trapezoidal or angular incision. The mucoperiosteal flap was turned up with a periosteal stripper to expose the lateral maxillary sinus. Then, at a distance of approximately 5-10 mm from the top of alveolar ridge, the bone plate of the lateral maxillary sinus was removed using a disc drill under cooling with normal saline to expose the light blue mucosa. Subsequently, the stripper was used to carefully peel the mucosa along the Schneiderian membrane and augment the height of the membrane. Next, a hemostatic gelatin sponge was mixed with autologous blood, autologous bone debris, and Bio-Oss bone particles. The bone-particle mixture was kept to coagulate into blocks, which were compacted between alveolar bones and implanted into the Schneiderian membrane. Holes were prepared step by step on the top of the alveolar ridge, and the implants were placed.

**Fig 1:**
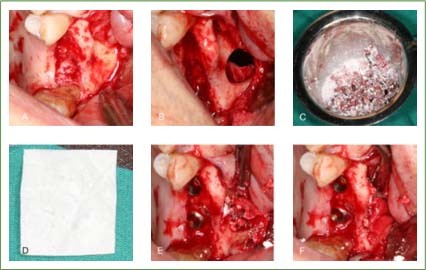
A representative case of maxillary sinus perforation. (A) Incision of the gingiva in the maxillary posterior tooth area, exposing the operation area. (B) Removal of the bone wall of the lateral maxillary sinus with a disk bur, peeling off the mucous membrane perforation. (C) Mixture of Bio-Oss bone particles, autologous blood, and hemostatic sponge. (D) Bio-Gide collagen membrane used in the operation. (E) The collagen membrane was placed between the Schneiderian membrane and the bone in the perforation area and filled with bone particles, then the implants were placed routinely. (F) Covering the exposed collagen membrane on the lateral wall to prevent the leakage of bone particles.

Finally, the Bio-Gide collagen membrane was covered on the fenestration of the lateral maxillary sinus, and the mucoperiosteal flap was aligned and tightly sutured. For patients with membrane perforation, the dissection of the Schneiderian membrane was expanded. Making used of the elasticity of the mucosa, the perforation range was reduced. Bio-Gide collagen membrane monolayer was utilised to cover the periphery of the perforation area for repair (Fig 1).

### Postoperative Interventions

Antibiotics and dexamethasone were postoperatively administered. For patients with membrane perforation, furosemide nasal drops and eucalyptol, limonene, and pinene enteric-coated soft capsules were prescribed. Patients were advised not to sneeze, blow their noses or cough violently. The sutures were removed after 7-10 days. At 6 months postoperatively, a two-stage implantation was performed under local anesthesia. After the soft tissues had healed, a conventional implant mold was taken, and the restoration was completed. Routine follow-up was performed 12 months postoperatively.

### Data Collection

The demographic and clinical data of patients were collected, including sex, age, height of residual alveolar bone, implant system, implant diameter, and implant length.

**Fig 2:**
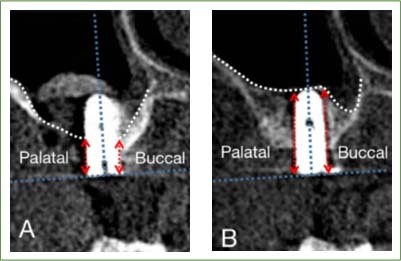
Measurement of vertical bone gain. (A) Baseline bone height on the day after the operation. (B) The vertical bone height was measured during follow-up.

Bone height was measured on cone-beam computed tomography (CBCT) immediately as well as 6 and 12 months after implantation. The mean distance between the buccal and palatal neck platform of the implant and the original maxillary sinus floor measured on the coronal plane of CBCT post-operatively was used as the baseline bone height (BBH) (Fig 2A). During follow-up, vertical bone height (VBH) referred to the mean distance between the buccal palatine side of the implant neck platform and the bottom of the maxillary sinus (Fig 2B). The difference between VBH and BBH during follow-up was defined as vertical bone gain (VBG). SMT was measured on CBCT before and 12 months after surgery. Marginal bone loss was measured at the mesial and distal sites of implants using periapical radiographs at 1 year after implant restoration.

Implant survival was defined as implant presence during follow-up. Implant failure was defined as implant loss or in need of removal. The percentage of surviving implants among the total number of implants was the implant survival rate. Postoperative complications consisted of facial swelling, nasal bleeding, intraoral bleeding, wound dehiscence and acute/chronic sinusitis, etc.

### Statistical Analysis

Statistical analysis was performed using SPSS 20.0 (IBM; Armonk, NY, USA). The continuous data with a normal distribution (according to the Shapiro-Wilk test) were presented as means ± SD and analysed using the independent-sample t-test. The continuous data with a skewed distribution were presented as median (IQR) and analysed using the Mann-Whitney U-test. The categorical data were presented as n (%) and analysed using the chi-squared test. Two-sided p-values <0.05 were considered statistically significant.

## RESULTS

### Baseline Data

Among the 60 enrolled patients, 30 cases (35 implants) of which 17 were male and 13 female, with an average age of 58.7±8.5 years, were allocated to the membrane perforation group, and 30 patients (44 implants) of which 15 were male and 15 female, aged 60.6±11.3 (mean), were assigned to the non-perforation group. There were no statistically significant differences in sex, age, the height of residual alveolar bone, implant system, implant diameter, and implant length between two groups (p>0.05) (Table 1).

**Table 1:** Baseline data

	Perforation group	Non-perforation group	p*
Number of patients	30	30	
Number of implants	35	44	
Sex			
Male (%)	17 (57.1%)	15 (50.0%)	0.639
Female (%)	13 (42.9%)	15 (50.0%)	
Age (years)	58.7±8.5	60.6±11.3	0.544
Implant system			
Bego S	24	25	0.260
Noble PMC	7	16	
Noble Active	4	3	
Implant diameter (mm)	4.2±0.3	4.2±0.3	0.765
Implant length (mm)	10.0±0.4	10.1±0.4	0.350
Implant survival rates	100%	100%	

*Chi-squared test or Student’s t-test.

### VBG

The mean vertical bone gain at 6 months postoperatively months in the perforation group was 6.02±2.14 mm compared to 6.78±2.59 mm in the non-perforation group, and 5.37±2.22 mm compared to 6.42±2.64 mm at 12 months postoperatively. No statistically significant differences were observed in the vertical bone gain at 6 and 12 months postoperatively between the two groups (p>0.05) (Table 2).

**Table 2:** Comparison of vertical bone gain in mm during follow-up between two groups (mean ± SD)

	Perforation group (n=35)	Non-perforation group (n=44)	p*
6 months	6.02±2.14	6.78±2.59	0.218
12 months	5.37±2.22	6.42±2.64	0.282

*Student’s t-test.

### SMT

The median SMT in the perforation group was 0.77 mm, significantly thinner than the 1.24 mm in the non-perforation group before surgery (p<0.05), but there was no statistically significant difference in median SMT at 12 months between the two groups (0.80 mm vs 1.25 mm, p>0.05; Table 3.)

**Table 3:** Comparison of Schneiderian membrane thickness in mm during follow-up between two groups (median (Q1–Q3])

	Pre-operation	12 months
Perforation group (n=35)	0.77 (0.66~1.01)	0.80 (0.60~0.95)
Non-perforation group (n=44)	1.24 (0.83~2.00)	1.25 (0.64~1.57)
p*	0.029	0.104

*Mann-Whitney U-test.

### Marginal Bone Loss

The marginal bone loss at 1 year after implant restoration in the perforation and non-perforation groups was 0.16±0.10 mm and 0.22±0.12 mm, with no statistically signicant difference (p>0.05).

### Implant Survival Rate

During postoperative follow-up, there was no loosening or loss of implants in either group, and the implant survival rate was 100% in both groups (Table 1).

### Postoperative Complications

In the perforation group, 15 patients developed varying degrees of epistaxis, and 18 patients suffered from facial swelling, whereas there was no secondary chronic maxillary sinusitis. In the non-perforation group, five patients presented with epistaxis, 19 cases of facial swelling, one case of intraoral bleeding, and two cases of chronic maxillary sinusitis, respectively. No postoperative reactions, such as wound dehiscence, severe pain or acute inflammation, were noted. Table 5 shows that the incidence of nasal bleeding in the membrane perforation group was 50% (15/30), significantly higher than 16.7% (5/30) in the non-perforation group (p<0.05).

**Table 4:** Comparison of marginal bone loss in mm 1 year after implant restoration between the two groups (mean ± SD, mm)

	Perforation group (n=35)	Non-perforation group (n=44)	p*
Marginal bone loss	0.16±0.10	0.22±0.12	0.324

*Student’s t-test.

**Table 5:** Postoperative complications

	Perforation group (n=30)	Non-perforation group (n=30)	p*
Facial swelling	18	19	0.791
Nasal bleeding	15	5	0.006
Intraoral bleeding	0	1	1.000
Wound dehiscence	1	0	1.000
Acute/chronic sinusitis	0	2	0.492

*Chi-squared test.

## DISCUSSION

When performing sinus floor elevation, the risk of perforation of the sinus membrane must be considered. In the systematic review by Pjetursson et al,^19^ the perforation rate ranged from 0 to 58.3%, with a mean of 19.5%. Nolan et al^16^ conducted a retrospective study consisting of 359 cases of external elevation, finding the perforation rate to be 41%. Although unexpected perforation of Schneiderian membrane occurs during external elevation, surgeons can perform perforation repair such as coverage of absorbable membrane, suturing under direct vision,^17,18,20^ which is highly efficaceous for small perforations. Recently, Park et al^17^ successfully directly filled the perforation with solidified bone substitute, simultaneously placed an implant and achieved good clinical results, offering a novel treatment option.

The Schneiderian membrane is composed of pseudostratified ciliated columnar epithelium, connective tissue, and periosteum to maintain the health and drainage of the Schneiderian membrane.^21^ Perforation causes oedema or bleeding, which may lead to obstruction of the maxillary sinus orifice and damage the drainage capacity of the nasal sinus mucous, resulting in acute or chronic maxillary sinusitis.^23^ A retrospective study by Schwarz et al^26^ showed a positive correlation between mucosal perforation and the occurrence of maxillary sinusitis. The incidence of maxillary sinusitis after perforation is approximately 31.4%. In the present study, the incidence of maxillary sinusitis after perforation was low, which might be associated with the use of a colloidal silver hemostatic sponge (Gelatamp, Coltene; Alstätten, Switzerland). Gelatamp has been widely used in preventing clinical complications, but also for site retention, and periodontal tissue regeneration, effectively contributing to clot formation. The sponges become soft after contact with blood.^13^ In this study, we innovatively combined Gelatamp use with Bio-Ossxx particles, providing the following advantages: the release of silver ions to kill bacteria and prevent infection, blood coagulation, and expansion to prevent continuous bleeding. Moreover, the Gelatamp sponge can be stably infiltrated by free bone particles in the blood and maintain the space of newly-formed bone. Nevertheless, two patients presented with chronic maxillary sinusitis in the non-perforation group, probably due to their history of chronic maxillary sinusitis; their final implant restoration was completed after otorhinolaryngology treatment. Tilaveridis et al^29^ observed that patients with a medical history of maxillary sinusitis had a higher risk of maxillary sinusitis after implantation. Timmenga et al^30^ found that patients with chronic maxillary sinusitis were more likely to develop maxillary sinusitis after augmentation.

Maintaining the integrity of the Schneiderian membrane is conducive to achieving better bone formation, as the membrane has potential osteogenic properties.^28^ In our study, the thickness of the maxillary sinus membrane in the perforated group before surgery was thinner than that in the non-perforated group, which can also be one of the reasons for perforation. However, after proper perforation repair, it seems to have little effect on bone formation and marginal bone loss. Proussaefs et al^20^ observed that bone formation in non-perforated sites was significantly higher than that in perforated sites. Furthermore, Froum et al^8^ showed that the perforation site could be repaired properly during the operation, and no adverse complications on osteogenesis were noted.

In this study, VBGs were used as the reference indices of bone formation. No statistically significant differences were observed between the perforation and non-perforation groups at 6 and 12 months postoperatively. There was a difference in the density between the original residual bone tissue and the newly-implanted bone substitute material on the day after implantation, and a dividing line could be observed. This dividing line can be utilised to determine a reference point when implanted immediately, which was the residual bone height of the above reference point. The distance between the BBH and VBH measured during follow-up can intuitively represent the reconstruction of bone substitute materials to some extent. Despite these outcomes, significant bone formation occurred in the membrane perforation group after the operation due to the expansion of bone substitute infiltration in tissue fluid and blood after implantation. Nevertheless, it was difficult to confirmed this by measurement, because the bone particles might leak into the maxillary sinus immediately post-perforation, and it was difficult to accurately determine the edge. Similarly, Huang et al^9^ showed that evident bone resorption was observed within 6 months after lateral augmentation and simultaneous implantation, indicating that bone formation and reconstruction occurred at the perforation. Park et al^17^ found that bone tissue after unrepaired lateral augmentation would gradually become stable. In the present study, although it is difficult to accurately measure the vertical bone height immediately after surgery, there was no statistically significant difference in the vertical height of new bone between the perforation and non-perforation groups during postoperative follow-up, indicating that the effect of perforation on bone formation was relatively small.

No consensus has been reached regarding the effect of Schneiderian membrane perforation on the survival rate of implants. Nolan et al^16^ found that the implant failure rate after perforation was statistically significantly higher than that in patients with an intact membrane. However, most studies have shown that the incidence of perforation does not affect the survival rate of implants.^1,2,11,18^ A recent meta-analysis^31^ consisting of seven studies proved that a total of 1115 implants were placed under the perforated and repaired membrane with a survival rate of 97.7%, and 2495 implants were placed under the undamaged sinus membrane, with a survival rate of 98.9%. In the present study, perforation did not statistically significantly impact the survival rate of the implant.

### Study limitations

Several limitations must be mentioned. This was a single-center study with a small sample size. The retrospective nature of this study confined to the data analyses that are available in the patient charts. In addition, the follow-up duation was relatively short; the middle- and long-term outcomes remain to be investigated by subsequent studies.

## CONCLUSIONS

Despite the risk of postoperative complications when Schneiderian membrane perforation occurred during simultaneous external elevation and implant placement, it did not adversely affect the short-term clinical and radiographic outcomes. In addition, it may be possible to maintain the integrity of the Schneiderian membrane during the operation.
